# Effect of chronic lymphocytic thyroiditis on the efficacy and safety of ultrasound‐guided radiofrequency ablation for papillary thyroid microcarcinoma

**DOI:** 10.1002/cam4.2406

**Published:** 2019-07-30

**Authors:** Yan Zhang, Ming‐bo Zhang, Yu‐kun Luo, Jie Li, Ying Zhang, Jie Tang

**Affiliations:** ^1^ Department of Ultrasound Chinese People's Liberation Army General Hospital Beijing China; ^2^ Department of Pathology Chinese People's Liberation Army General Hospital Beijing China

**Keywords:** ablation, contrast media, radiofrequency, thyroid carcinoma; ultrasonography

## Abstract

**Background:**

Chronic lymphocytic thyroiditis (CLT) is an autoimmune disease commonly associated with papillary thyroid carcinoma characterized by a smaller primary tumor size at presentation. The efficacy and safety of ultrasound‐guided radiofrequency ablation (RFA) for papillary thyroid microcarcinoma (PTMC) coexisting with CLT is still unknown.

**Methods:**

Sixty patients with unifocal PTMC were enrolled and classified into PTMC and PTMC+CLT groups (n = 30/group). CLT was diagnosed histopathologically. The ablation area exceeded the tumor margins, and was evaluated by US and contrast‐enhanced US (CEUS) for residual tumor to prevent recurrence. Three months after ablation, US‐guided core‐needle biopsy was performed to assess the presence of residual and recurrent cancer. Preoperative and postoperative data on patients and tumors were recorded and analyzed.

**Results:**

There were no differences between groups in age, sex, preoperative tumor volume, ablation time, or ablation power (*P > *0.05). There was also no significant difference in postoperative ablation zone volume between the groups at the 1‐, 3‐, 6‐, 12‐, and 18‐month follow‐ups (*P* > 0.05). The volume reduction rate significantly differed between the two groups at month 3 (*P* = 0.03). The ablation area could not be identified on US and CEUS at 9.8 ± 5.0 and 10.0 ± 4.8 months in the PTMC and PTMC + CLT groups, respectively (*P* = 0.197). No serious complications occurred during and after ablation. No residual cancer cells were found on biopsy after ablation.

**Conclusions:**

RFA was effective in patients with PTMC+CLT, and its therapeutic efficacy and safety were similar to those in patients with PTMC without CLT.

## 1 INTRODUCTION

Since the mid‐1990s, the incidence of thyroid cancer has increased worldwide and is reportedly the fastest‐growing cancer. In the United States, the overall incidence of thyroid cancer increased by 3% annually from 1974 to 2013^1^ and the number of new cases likely reached 53 900 in 2018.^2^ In China, the incidence of thyroid cancer is also increasing, and in 2014, it was among the top four cancers in terms of incidence among women.^3^ Thyroid cancer includes several pathological types, of which papillary thyroid carcinoma (PTC) is the most common. Tumors ≤10 mm in diameter are defined as papillary thyroid microcarcinomas (PTMCs). These have been observed in 15.5% of autopsies in which whole‐gland examination was performed and are typically associated with good prognosis.^4^

Chronic lymphocytic thyroiditis (CLT), also known as Hashimoto's thyroiditis, is an autoimmune disease characterized by widespread lymphocyte infiltration, fibrosis and parenchymal atrophy of the thyroid tissue. The male‐to‐female incidence ratio of CLT is 1:5‐20. CLT is commonly associated with PTC, and is characterized by a smaller primary tumor size at presentation. Leni et al^5^ reported that one‐third of PTC cases (33.3%, 168/505) have CLT coexistence. No association has been found between CLT and follicular, medullary, or anaplastic thyroid cancer.^6^ The coexistence of PTC and CLT is reportedly associated with better prognoses, lower rates of lymph node and distant metastases, and recurrence, particularly in patients aged ≥45 years.^7^ There is controversy surrounding the treatment of PTC with coexisting CLT. For PTMC, some guidelines recommend the performance of surgery.^8^, ^9^ However, traditional surgery results in injuries, prominent neck scarring, and a lowered quality of life, especially among older patients with chronic comorbidities. The Korean Society of Thyroid Radiology consensus statement^10^ highlights the need for active surveillance rather than immediate surgery in adult patients with low‐risk PTMC. However, a majority of patients experience severe anxiety if no treatment is provided. Therefore, minimally invasive therapy is utilized in many cases. Thermal tumor ablation (using microwave, radiofrequency, or laser ablation, or high‐intensity focused ultrasound [US]) has been applied in clinical practice with good feedback.^11^, ^12^, ^13^ Radiofrequency ablation (RFA) was found to be efficient and safe in the treatment of PTMC in our preliminary study.^14^ We aimed to investigate the therapeutic effect and safety of RFA in PTMC cases with CLT in this study.

## 2 MATERIALS AND METHODS

This study was approved by the ethics committee of our hospital, and informed consent was obtained from each patient before the performance of US‐guided core‐needle biopsy (CNB) and RFA. The informed consent form for RFA emphasized that surgery is the routine treatment procedure recommended by guidelines, and that RFA administration cannot prevent the development of recurrent PTMC and undetectable cervical lymph node metastasis (LNM).

### 2.1 Patients

Patients who fulfilled the following criteria were enrolled: (1) presence of PTC confirmed by US‐guided CNB; (2) maximum diameter less than 1 cm; (3) absence of capsular infiltration and extrathyroidal invasion, and lack of LNM detection; (4) absence of neck irradiation history; and (5) unable or refused to receive surgery. Exclusion criteria were: the presence of (1) multifocal cancer; (2) aggressive histological PTMC, such as tall cell, insular, or columnar cell carcinoma; (3) suspicious cervical LNM; (4) pregnancy and lactation; (5) severe coagulation disorders, respiratory failure, myocardial infarction, systemic infection, or uncontrolled diabetes; (6) neuropsychiatric disturbance or neck extension disorder leading to RFA nontolerance; (7) cardiac pacemaker implantation; (8) contralateral vocal cord paralysis; and (9) allergic to sulfur hexafluoride microbubbles (SonoVue, Bracco International, Milan, Italy).

Between February 2013 and March 2017, 60 tumors in 60 patients (12 men and 48 women) treated with US‐guided RFA were included, comprising 30 patients with only PTMC, and 30 with CLT+PTMC. CLT was confirmed by pathologic examination and serological test, and was defined as diffuse lymphocytic and plasma cell infiltrates, lymphoid follicles formation with germinal centers, varying degree of fibrosis, parenchymal atrophy, and the presence of large follicular cells with oxyphilic cell changes,^7^ as well as positive hyroperoxidase antibody (TPOAb) and thyroglobulin antibody (TgAb). The presence of peritumoral inflammatory reaction was not considered CLT. All patients had complete records and were followed for more than 18 months.

### 2.2 Instrument and equipment

US and contrast‐enhanced US (CEUS) examinations were performed using a Siemens Acuson Sequoia 512 Ultrasound System (Siemens, Mountain View, CA) with a 15L8W linear array transducer. US‐guided RFA and CNB were performed using a Siemens Acuson Sequoia 512 Ultrasound System with a 6L3 linear array transducer. CNB on each nodule was performed using an 18‐gauge biopsy needle after RFA (Biopty; Bard, Covington, GA).

A bipolar RFA generator (CelonLabPOWER; Olympus Surgical Technologies Europe, Hamburg, Germany) and an 18‐gauge bipolar radiofrequency (RF) applicator with a 0.9‐cm active tip (CelonProSurge micro 100‐T09; Olympus Surgical Technologies Europe) were used for RFA treatment in this study. During the application of RF energy, the electric impedance of the tissue between the two electrodes at the tip of the RF applicator was measured continuously by the generator. The power is automatically reduced if the temperature at the electrodes reaches 100°C.

### 2.3 Preablation assessment

Careful history‐taking and thorough physical examinations were conducted in all patients in our department. All patients who qualified for RFA were subject to thyroid US, CEUS, and determination of the levels of free thyroid hormones T3 (normal reference range: 2.76‐6.30 pmol/L) and T4 (10.42‐24.32 pmol/L), thyroid‐stimulating hormone (TSH) (0.35‐5.5 mU/L), TPOAb (＜60 IU/mL) and TgAb (＜60 IU/mL).

Patients were supine with the neck extended during the procedure. An intravenous line was introduced into an elbow vein. US appearances were evaluated and recorded according to the multidisciplinary consensus statement for thyroid nodules.^15^ For each tumor, the size, volume, location, echogenicity, margin, shape (height/width), calcifications, and vascularity were evaluated by US. The volume of each tumor was calculated as V = πabc/6 (V: volume, a: transverse diameter of tumor, b: vertical diameter of tumor, and c: anteroposterior diameter of tumor). CEUS with a low mechanical index (0.19‐0.24) was used to describe the blood supply region of the tumor before and after RFA. The contrast agent used was 59 mg of dry powder SonoVue constituted in 5 mL of normal saline. CEUS was performed after a bolus injection of SonoVue (2.4 mL), followed by a normal saline flush (5 mL). Real‐time microbubble perfusions within the tumor and surrounding tissues were observed for a minimum of 2 minutes and recorded electronically.

Capsular and extrathyroidal invasion of thyroid cancer were evaluated by both US and CEUS. Extracapsular extension on the US image was defined as cases in which the anterior and posterior hyperechoic thyroid capsules were discontinued. During the real‐time and multi‐angle scanning, the capsular infiltration and extrathyroidal invasion on CEUS were shown as low‐ or nonenhancing areas on the thyroid capsule invaded by malignant nodules.^16^

### 2.4 Ablation procedure

Conventional US was performed to evaluate the relationship between the tumor and critical structures in the neck, such as the trachea, esophagus, jugular vein, common carotid artery (CCA), and recurrent laryngeal nerves, in order to decide the best insertion way. A local anesthetic (1% lidocaine) was injected at the subcutaneous puncture site and the thyroid anterior capsule. If the distance between the tumor and critical neck structures was <5 mm, normal saline was injected using another needle (23 gauge) to form at least a 1cm distance between the tumor and critical structure for the prevention of thermal injuries. RFA was performed using the moving‐shot technique (P–Q); 3W was the initial radiofrequency power which was increased to 5W if a transient hyperechoic zone did not form at the electrode tip within 5‐10 s. To prevent residual tumor and recurrence, the RFA area exceeded the tumor margin. The ablation procedure ended when the tumor was completely covered with hyperechoic zone in three‐dimensional space on US. The damage range of ablation was evaluated by CEUS immediately after ablation, and the electrode needle was pulled out of the tissue after ensuring that no residual tumor (completely no enhancement in the ablation area). If the enhancement was showed in some areas of tumor, complementary ablation could be applied promptly to avoid residual cancer cells. During the procedure, special attention was given to the protection of critical neck structures to prevent the significant complications such as hematoma or nerve injury. All complications occurring during and after RFA were carefully assessed according to patients' clinical signs and symptoms. Each patient was observed for 1‐2 hours after RFA.

### 2.5 Follow‐up

Patients were followed‐up using conventional US and CEUS at months 1, 3, 6, and 12 after RFA, and every 6 months thereafter. The ablation area was evaluated by CEUS to detect volume of ablation and provide a baseline to screen for recurrence. The volume reduction ratio (VRR) was calculated as follows: VRR (%) = [(1 − final volume)/initial volume] × 100%. The involvement of cervical lymph nodes was evaluated by US; suspicious lymph nodes (a globular shape, loss of the normal echogenic hilum, presence of peripheral rather than hilar flow, and microcalcifications^17^) were biopsied. US‐guided CNB was performed at the center and the margin of the ablation area, and in the surrounding thyroid parenchyma 3 months after RFA. Complications during the follow‐up period were assessed according to the reporting standards of the Society of Interventional Radiology.^18^

All RFA procedures and preoperative examinations were performed by one experienced physician to exclude bias associated with different operators. Postoperative CNB was also performed by the same physician who performed RFA. Follow‐up conventional US and CEUS examinations were performed by two other experienced physicians who were blinded to the histological and imaging findings. Discrepancies were resolved by the judgment of a third experienced physician who had specialized in thyroid US and CEUS for over 15 years.

### 2.6 Statistical analysis

All statistical analyses were performed using SPSS software package, Version 13 for Windows (SPSS Inc, Chicago, IL). A chi‐squared test (χ^2^) was used to analyze the categorical variables. Continuous data were reported as mean ± standard deviation (range). Volume and VRR of the ablation area before RFA and at each follow‐up were analyzed by the *t* test. The Wilcoxon signed rank test was used to compare tumor calcification, color Doppler flow imaging (CDFI) blood flow grades, and changes in the number of patients with tumor disappearance at each follow‐up between CLT+PTMC and PTMC groups. The Wilcoxon rank sum test was used to compare Free T3, T4, and TSH values in patients with and without CLT *P* values <0.05 were considered statistically significant.

## 3 RESULTS

### 3.1 Preoperative patient and tumor characteristics

There were no significant differences in terms of age, sex, and tumor volume between the PTMC and PTMC+CLT groups (*P* > 0.05) (Table 1). All thyroid nodules were hypoechoic; other characteristics are shown in Table 2. There were no significant differences in nodule location, margin, shape, height/width, calcification, and CDFI type between the two groups (*P* > 0.05). The distance between the nodule and trachea or common carotid artery (CCA) was <2 mm in 13 nodules, including eight near the trachea (0.117 ± 0.024) cm, and five near the CCA (0.094 ± 0.013) cm. There were no significant differences in free T3, T4 and TSH values between the two groups (*U* = 0.434, *P* = 0.664; *U* = 0.452, *P* = 0.651; and *U* = 0.886, *P* = 0.376, respectively). All patients with CLT had positive TgAb and TPOAb. The thyroid function test results of those with PTMC were in the normal range.

**Table 1:** Comparison of the preoperative data of the patients and tumor volume between the PTMC+CLT and PTMC groups

	PTMC+CLT	PTMC	*T* (χ^2^)	*P*
Age	42.2 ± 9.61	44.07 ± 8.99	0.78	0.44
Sex				
Male	4	8	1.67	0.20
Female	26	22		
Tumor volume (mm^3^)	0.10 ± 0.13	0.11 ± 0.17	1.67	0.75

Abbreviations: CLT, chronic lymphocytic thyroiditis; PTMC, papillary thyroid microcarcinoma.

**Table 2:** Ultrasonic characteristics of the tumors before RFA in the PTMC+CLT and PTMC groups

	PTMC+CLT	PTMC	χ^2^ *(U)*	*P*
Location				
Inner side	5	11	3.15	0.37
Lateral side	7	5		
Center	16	12		
Isthmus	2	2		
Margin				
Defined	9	14	1.76	0.18
Ill‐defined	21	16		
Shape				
Regular	8	6	0.37	0.54
Irregular	22	24		
Height/width				
>1	10	13	0.63	0.43
≤1	20	17		
Calcification				
Macrocalcification	2	2	3.15	0.21
Microcalcification	5	11		
No calcification	23	17		
CDFI				
Type I	21	18	0.66	0.42
Type II	9	12		
Type III	0	0		
Total		30	30	

Abbreviations: CDFI, color Doppler flow imaging; CLT, chronic lymphocytic thyroiditis; PTMC, papillary thyroid microcarcinoma.

### 3.2 Ablation power and time in the PTMC and PTMC+CLT groups

The ablation powers in the PTMC and PTMC+CLT groups were 0.9 ± 0.5 KJ and 0.7 ± 0.5 KJ, respectively (*t* = 1.453, *P* = 0.1515); ablation times were 3.8 ± 2.1 min and 2.9 ± 2 0.1 min, respectively (*t* = 1.801, *P* = 0.0768).

### 3.3 Tumor volume and VRR after RFA

**Figure 1:**
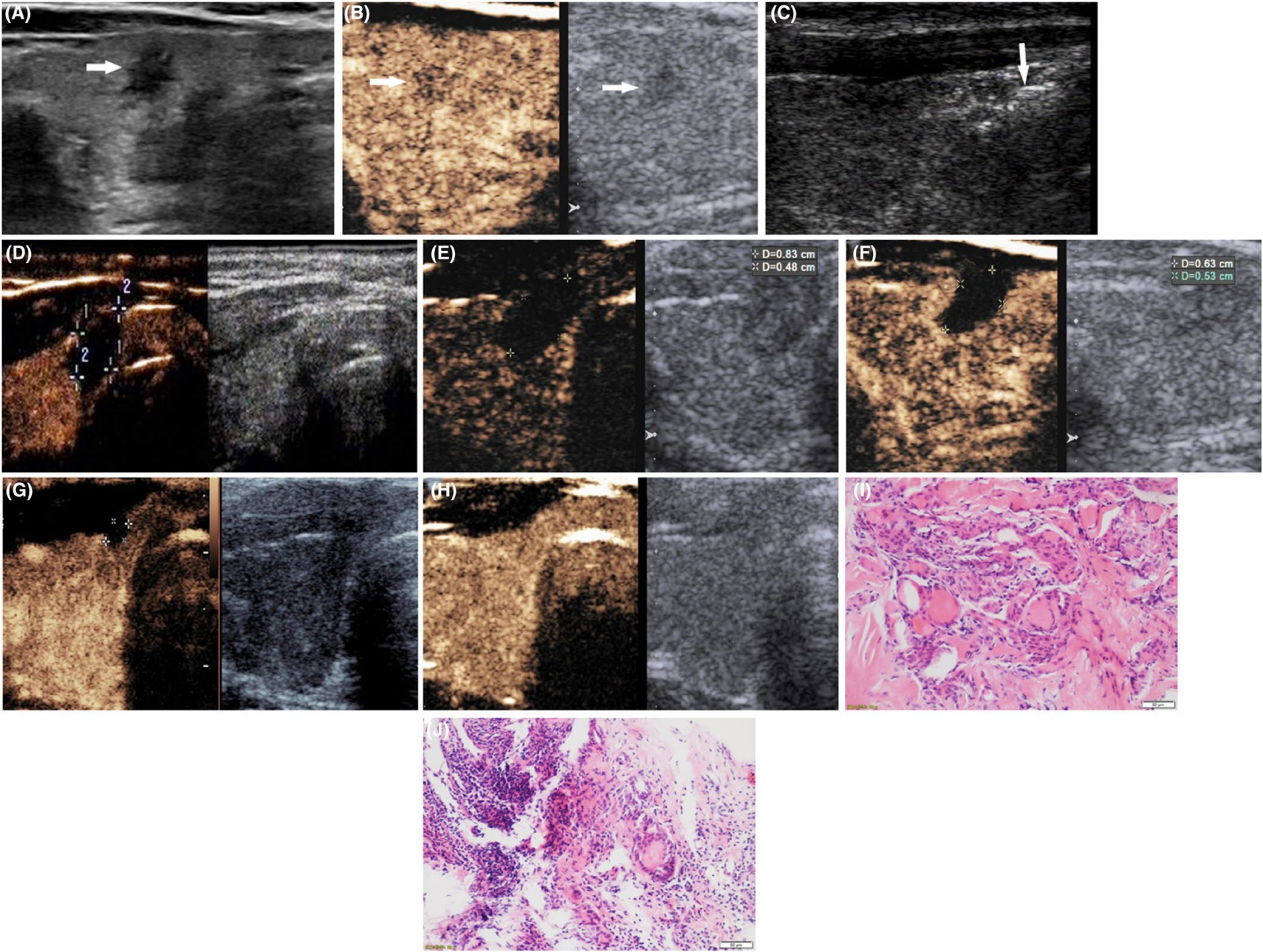
Radiofrequency ablation (RFA) treatment and follow‐up of one case of papillary thyroid microcarcinoma with chronic lymphocytic thyroiditis. (A) A hypoechoic nodule sized 0.4 × 0.5 × 0.4 cm, with irregular margins and microcalcifications was displayed in the right thyroid lobe (arrow). (B) Uneven and irregular hypo‐enhancement in the nodule was observed by contrast‐enhanced ultrasound (CEUS) (arrow, left image). (C) During RFA, the nodule was covered by a hyperechoic area (arrow) on US. (D) Immediately after RFA, the ablation area was showed completely no enhancement by CEUS, and its size (0.7 × 1.1 × 1.0 cm) was larger than the initial nodule size. (E) One month after RFA, the ablation area decreased in size to 0.9 × 0.8 × 0.5 cm. (F) Three months after RFA, the ablation area decreased to 0.6 × 0.5 × 0.6 cm. (G) Six months after RFA, the ablation area decreased to 0.3 × 0.2 × 0.3 cm. (H) The ablation area could not be identified on both US and CEUS. (I) Before RFA, the pathologic examination of this nodule showed the presence of papillary thyroid carcinoma accompanied by chronic lymphocytic thyroiditis. (J) Three months after RFA, pathology showed degenerated and necrotic follicular epithelia, interstitial fibrous tissue hyperplasia, and hyaline degeneration in the ablation lesion, with lymphocyte infiltration and multinucleated giant cell reaction in the adjacent thyroid tissue. No residual cancer was found

**Figure 2:**
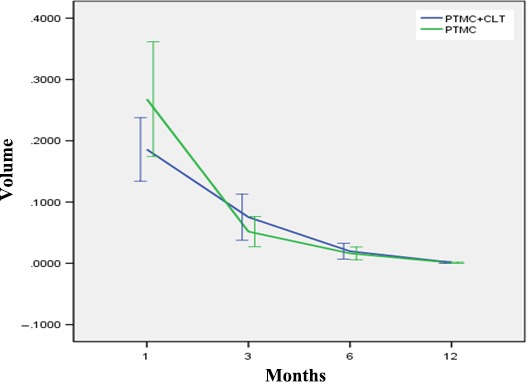
Changes in ablation zone volume in PTMC cases with and without CLT at each follow‐up. PTMC, papillary thyroid microcarcinoma; CLT, chronic lymphocytic thyroiditis

In terms of postoperative ablation volume, there was no significant difference between the two groups at 1, 3, 6, 12, and 18 months after RFA (*P* > 0.05) (Table 3, Figure 1). While there was a significant difference in VRR between the two groups 3 months after ablation (*t* = 1.28, *P* = 0.03), no difference was observed at any other time‐point (*P* > 0.05) (Table 4, Figure 2).

**Table 3:** Changes in the tumor volume between the PTMC + CLT and PTMC groups after RFA and at each follow‐up

Time	PTMC+CLT (mm^3^)		PTMC (mm^3^)		*T*	*P*
	M ± SD	Range	M ± SD	Range		
Immediately	0.81 ± 0.56	0.15‐2.37	0.93 ± 0.50	0.17‐1.86	0.91	0.37
1 mo	0.19 ± 0.14	0.05‐0.60	0.27 ± 0.25	0.03‐1.20	1.57	0.12
3 mo	0.08 ± 0.10	0‐0.41	0.05 ± 0.07	0‐0.21	1.07	0.29
6 mo	0.02 ± 0.04	0‐0.15	0.02 ± 0.03	0‐0.11	0.43	0.67
12 mo	0.001 ± 0.003	0‐0.02	0.001 ± 0.004	0‐0.01	0.49	0.62
18 mo	0	0	0	0	0.00	1.00

Abbreviations: CLT, chronic lymphocytic thyroiditis; M, mean; PTMC, papillary thyroid microcarcinoma; RFA, radiofrequency ablation; SD, standard deviation.

**Table 4:** Changes in the tumor volume reduction ratio between the PTMC+CLT and PTMC groups after RFA and at each follow‐up

Time	PTMC+CLT (%)		PTMC (%)		*T*	*P*
	M ± SD	Range	M ± SD	Range		
1 mo	0.70 ± 0.21	0.13‐0.98	0.73 ± 0.15	0.33‐0.93	0.46	0.65
3 mo	0.90 ± 0.11	0.53‐1	0.95 ± 0.07	0.71‐1	2.18	0.03
6 mo	0.97 ± 0.04	0.83‐1	0.99 ± 0.03	0.90‐1	1.33	0.19
12 mo	0.998 ± 0.007	0.97‐1	0.999 ± 0.003	0.99‐1	0.61	0.55
18 mo	1	1	1	1	0	1

Abbreviations: CLT, chronic lymphocytic thyroiditis; M, mean; PTMC, papillary thyroid microcarcinoma; RFA, radiofrequency ablation; SD, standard deviation.

After RFA, the times to tumor disappearance were 9.800 ± 5.041 months and 10.0 ± 4.8 months (*t* = 0.16, *P* = 0.88) in the PTMC and PTMC + CLT groups, respectively. A total of 43% of the nodules in the PTMC+CLT group resolved in 12 months, and 47% in the PTMC group resolved in 6 months. No significant differences were observed with respect to the number of ablation areas between the groups (*u* = 0.319, *P* > 0.05) (Table 5). Calcification was observed in 20 PTMC cases, with time to tumor disappearance of 10.2 ± 5.1 months; no calcification was found in 40 PTMC cases, with time to tumor disappearance of 9.8 ± 4.9 months; no significant difference in time was observed between the PTMC cases with and without calcification (*t* = 0.28, *P* = 0.78). No residual cancer cells were found by CNB 3 months after ablation (Figure 1). No recurrent tumors or suspicious metastatic lymph nodes were detected.

**Table 5:** Number of patients with tumors disappearance in the PTMC+CLT and PTMC groups after RFA and at each follow‐up

Time	PTMC+CLT (N, %)	PTMC (N, %)	*U*	*P*
3 mo	4 (13.33%)	2 (6.67%)	0.32	＞0.05
6 mo	8 (26.67%)	14 (46.67%)		
12 mo	13 (43.33%)	8 (26.67%)		
18 mo	5 (16.67%)	6 (20%)		
Total	30	30		

Abbreviations: CLT, chronic lymphocytic thyroiditis; PTMC, papillary thyroid microcarcinoma; RFA, radiofrequency ablation.

### 3.4 Complications

Slight voice hoarseness was observed in one patient (1.7%, 1/60) after RFA; voice recovery occurred without treatment in 1 week. The presence of moderate‐intensity pain was reported by two patients (3.3%, 2/60), and slight fever was noted in one patient (1.7%, 1/60); in all cases, spontaneous recovery was observed.

## 4 DISCUSSION

The feasibility of RFA in thyroid cancer treatment is still controversial, as some surgeons believe that it is difficult to detect tiny lymph node metastases using US^19^; in addition, the use of prophylactic central compartment neck dissection (CCND) is more reassuring for patients with clinically node‐negative PTC. However, with the increasing incidence of thyroid cancer, more patients with low‐risk microcarcinomas are receiving unnecessary extended thyroidectomy and prophylactic CCND. Previous studies have shown that the proportion of CCND with negative findings ranges from 57.6% to 84.8%,^20^, ^21^ and that excessive surgery decreases patients' quality of life (postoperative complications included transient hypoparathyroidism, permanent hypoparathyroidism, vocal cord palsy, and bleeding,). Therefore, the treatment approach for this disease is changing. In recent years, prophylactic CCND has not been recommended for PTMC patients without LNM.^8^, ^22^, ^23^, ^24^, ^25^ The median risk of local‐regional lymph node recurrence varies markedly by clinical staging in patients with pathologically proven neck LNM, with recurrence rates of 2% (range 0%‐9%) in patients with an initial N0 stage vs 22% (range 10%‐42%) in those with initially positive lymph nodes. Furthermore, the median risk of recurrence in LNM patients varies markedly by the number of positive nodes, with values of 4% (range 3%‐8%) in cases with <5 nodes and 19% (range 7%‐21%) in those with >5 nodes.^23^ At the 5‐year follow‐up, no difference was observed in the outcomes of patients treated with total thyroidectomy and those treated with total thyroidectomy + prophylactic CCND.^24^ The results of these studies support the use of RFA for PTMC.

The presence of multifocality and capsular infiltration indicates a high risk of cancer invasion and metastasis.^25^, ^26^ Therefore, cases with a single tumor in the thyroid parenchyma and within the thyroid capsule were eligible for RFA in this study. Our preliminary study showed that RFA is efficient with a low complication rate in PTMC treatment.^14^ CLT is a type of chronic inflammation, and its effect on the ability to recover from the heat damage caused by RFA is still unknown.

No differences were observed in the preoperative volume and US characteristics of the tumors between the PTMC and PTMC+CLT groups (*P* > 0.05), and the patients' age and sex did not differ between the two groups (*P* > 0.05). Our results showed that recovery after ablation in patients with PTMC+CLT is similar to that in patients with only PTMC. The ablation volume in the PTMC group decreased rapidly in the first 3 months, and the VRR in the PTMC group was greater than that in the PTMC+CLT group (*P* = 0.03). Tissue damage due to trauma induces acute inflammation. The features of acute and subacute inflammation include the expansion of blood vessels (vasodilation), increase in blood flow (hyperemia), capillary permeability, and migration of neutrophils into the damaged tissue. However, the composition of white blood cells changes rapidly, with macrophages and lymphocytes replacing neutrophils. The hallmark of chronic inflammation is the infiltration of the primary inflammatory cells into the site, leading to the production of inflammatory cytokines, growth factors, and enzymes, thereby leading to progression of tissue damage and secondary repair processes, such as fibrosis and granuloma formation.^27^ The thyroid parenchyma in the case of CLT is already infiltrated by diffuse chronic inflammatory cells, so inflammation of the ablation zone was more marked in the acute and subacute periods after RFA. Immune cells were activated to phagocytize and remove the necrotic tissue, activating the autoimmune system so we saw no significant difference in ablation zone outcomes between the PTMC and PTMC+CLT groups. Three months after ablation, US‐guided CNB was performed at the center and margin of the ablation zone and in the adjacent thyroid parenchyma; pathological results showed degenerated and necrotic follicular epithelia, interstitial fibrous tissue hyperplasia, and hyaline degeneration in the central and peripheral areas of the ablation lesion, with lymphocyte infiltration and multinucleated giant cell reaction in the adjacent thyroid tissue. No residual cancer was found. Previous studies^28^, ^29^ with larger sample size showed that PTC with CLT has a good prognosis, and that the recurrence rate was lower than that associated with only PTC. This may be attributed to the fact that inflammation inhibits cancer‐cell proliferation. CLT may be involved in the destruction of cancer cells that express thyroid‐specific antigens in PTC as a result of its autoimmune response to the thyroid‐specific antigens. Few studies^30^ have shown no relationship between CLT and PTC. This study indicated that RFA is efficient and safe for PTMC cases with CLT; CLT should not be regarded as a factor that excludes a patient from receiving RFA.

In terms of safety, adhesions are found between the thyroid and adjacent connective tissue and muscles after ablation, distorting local anatomy, which results in difficulty performing surgery after ablation; therefore some surgeons have a negative attitude towards ablation. However, liquid isolation zone injection can be utilized to separate the thyroid from critical neck structures; this method is effective in preventing the occurrence of significant complications such as hematoma, and tracheal and nerve injury. Postoperative examination by both US and CEUS has shown clear boundaries of the thyroid gland and surrounding structures without signs of adhesion. In this study, 13 PTMC cases in which the distance to the trachea or CCA was <2 mm were successfully treated with RFA as a result of liquid isolation zone injection without serious complications.

This study had the limitation of a relatively short follow‐up period; longest follow‐up was 4 years and the shortest 20 months. The outcomes of all these patients should be confirmed in studies with larger sample size and extended follow‐up period.

In conclusion, this study found that RFA was effective and safe in PTMC+CLT patients. As evidenced by the low recurrence and high survival rates of these patients, CLT may be regarded as a protective factor for patients enrolled in PTMC treatment using RFA. The present study provides a basis for the study of immune regulation mechanisms induced by thyroid cancer necrosis.

## CONFLICT OF INTEREST

The authors made no disclosures.

## AUTHOR CONTRIBUTIONS

Yan Zhang contributed to the collection, analysis, and interpretation of data and writing initial draft. Mingbo Zhang performed the statistical analyses, had full access to all data, and takes responsibility for the accuracy of the data analysis in the study. Ying Zhang contributed to the collection, analysis and interpreted the results. Jie Li: contributed to the pathological figures analysis and interpretation. Jie Tang contributed to writing–review and revisions. Yukun Luo contributed to the study design, writing–initial draft, guarantor of the study and had full access to all data in the study, and takes responsibility for the integrity of the data and the accuracy of the data analysis. All authors contributed to critical revisions and approved the final version.
